# Correction: Robo2 regulates synaptic oxytocin content by affecting actin dynamics

**DOI:** 10.7554/eLife.63695

**Published:** 2020-10-05

**Authors:** Savani Anbalagan, Janna Blechman, Michael Gliksberg, Ludmila Gordon, Ron Rotkopf, Tali Dadosh, Eyal Shimoni, Gil Levkowitz

Anbalagan S, Blechman J, Gliksberg M, Gordon L, Rotkopf R, Dadosh T, Shimoni E, Levkowitz G. 2019. Robo2 regulates synaptic oxytocin content by affecting actin dynamics. *eLife* **8**:e45650. doi: 10.7554/eLife.45650.

Published 10, June 2019

In the published article, Figure 6F inadvertently presented the wrong confocal Z-stack image of Tg(oxt:Gal4) and Tg(UAS:mCherry-Utrophin-CH) expression in the neurohypophysis of 5-dpf transgenic larvae. The corrected Figure 6 is now shown. No further changes were made to the text and figure legend. Please note that this correction does not affect the results and conclusions of the original paper.The corrected Figure 6 is shown here:

**Figure 1:**
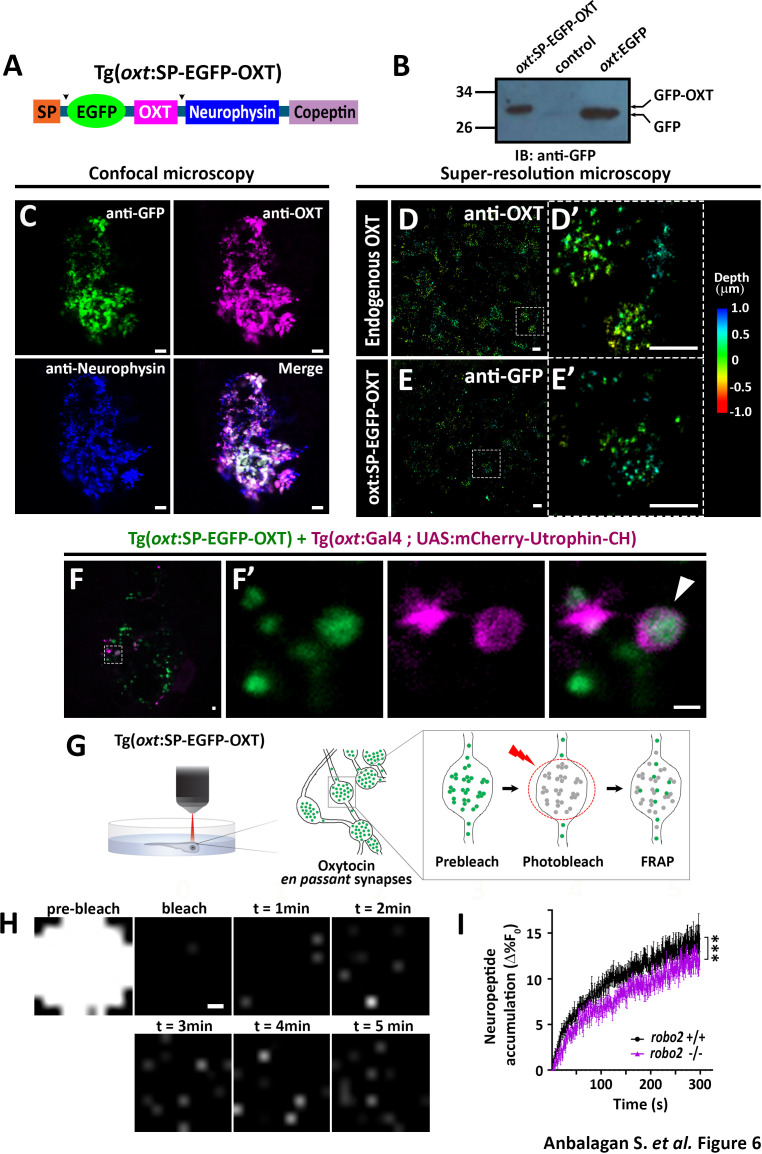


The originally published Figure 6 is also shown for reference:

**Figure 2:**
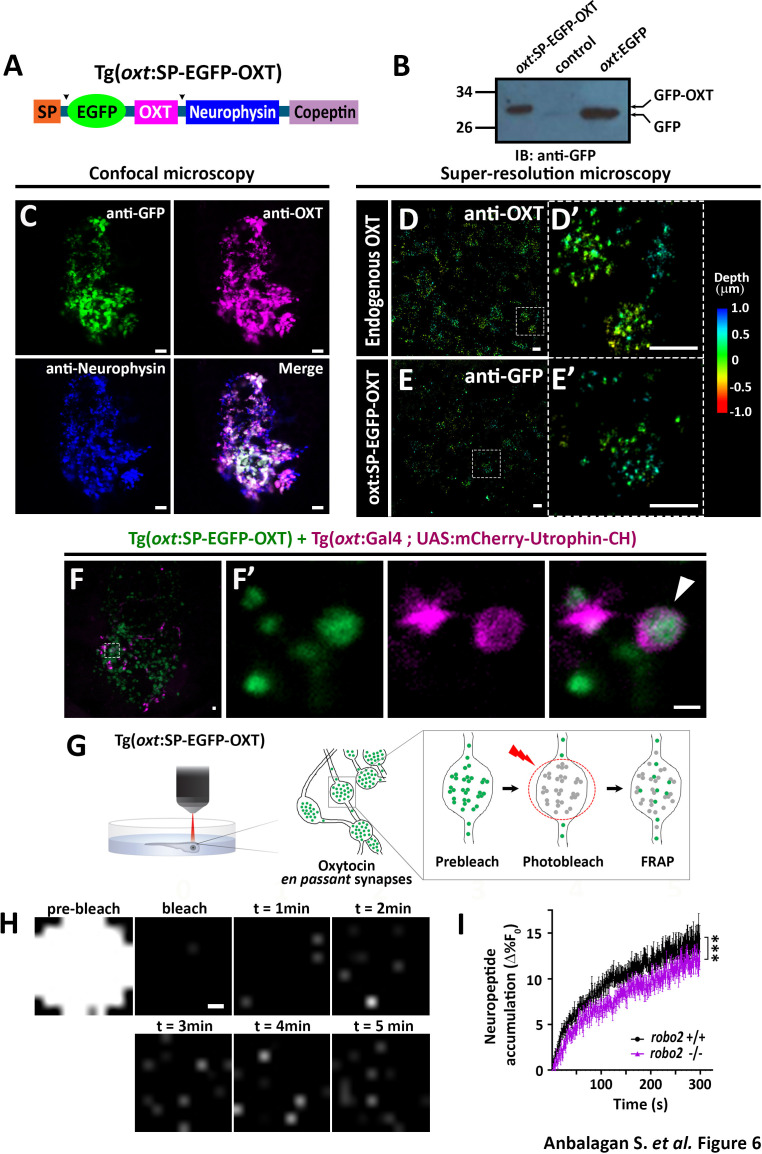


The article has been corrected accordingly.

